# Predictive Modeling of Antioxidant Coumarin Derivatives Using Multiple Approaches: Descriptor-Based QSAR, 3D-Pharmacophore Mapping, and HQSAR

**DOI:** 10.3797/scipharm.1208-01

**Published:** 2012-09-09

**Authors:** Indrani Mitra, Achintya Saha, Kunal Roy

**Affiliations:** 1 Drug Theoretics and Cheminformatics Laboratory, Department of Pharmaceutical Technology, Jadavpur University, Kolkata 700032, India.; 2 Department of Chemical Technology, University of Calcutta, 92 A P C Road, Kolkata 700 009, India.

**Keywords:** Antioxidant, QSAR, Pharmacophore, HQSAR

## Abstract

The inability of the systemic antioxidants to alleviate the exacerbation of free radical formation from metabolic outputs and environmental pollutants claims an urgent demand for the identification and design of new chemical entities with potent antioxidant activity. In the present work, different QSAR approaches have been utilized for identifying the essential structural attributes imparting a potential antioxidant activity profile of the coumarin derivatives. The descriptor-based QSAR model provides a quantitative outline regarding the structural prerequisites of the molecules, while 3D pharmacophore and HQSAR models emphasize the favourable spatial arrangement of the various chemical features and the crucial molecular fragments, respectively. All the models infer that the fused benzene ring and the oxygen atom of the pyran ring constituting the parent coumarin nucleus capture the prime pharmacophoric features, imparting superior antioxidant activity to the molecules. The developed models may serve as indispensable query tools for screening untested molecules belonging to the class of coumarin derivatives.

## Introduction

Free radical attack on the human system poses a vital threat to humankind. Most of the incurable and chronic diseases are the outcomes of an overload of free radical attacks in the human system resulting in an oxidative stress-like condition [1–4]. Free radicals are produced inevitably during cellular metabolism, such as by electron leakage from the electron transport chain and redox enzymes as well as by some lymphocytes while defending the human system against foreign organisms [1]. The susceptible lipid molecules present in the biological membrane are most prone to free radical attack. Additionally, DNA and protein-like macro molecules also constitute vulnerable targets for free radical attack. Free radical mutates the DNA and RNA by pairing with electrons in the DNA chains leading to cellular electronic imbalance [2]. Aging being characterized by the accumulation of mitochondrial DNA mutations, improper clearance of reactive oxygen species (ROS) produced by the respiratory chain results in early aging. Impaired lysosomal degradation of the free radical-damaged mitochondria also contributes to aging [3]. The protein molecules are functionally inactivated and subsequently degraded as a result of the free radical attack. When proteins critical for rapid homeostatic mechanisms (transport proteins) are affected by free radicals or when the proteolytic defense and/or other antioxidant defenses are overwhelmed, toxic events may ensue [4]. Such events constitute chronic pathologies such as artherosclerosis and chronic inflammatory diseases followed by rapid lyses of cells [5]. Besides these, the cells that line the arteries, the fat cells in the blood, and even the immune cells are largely affected by free radicals leading to the incidence of fatal degenerative diseases including cancer, cardiovascular disease, cataracts etc. [6, 7].

Antioxidants are now being fabricated as essential candidates to counteract these diseases [8]. The systemic antioxidants often fall short in maintaining the ROS balance either due to ineffective ROS clearance or due to excessive ROS formation. Such a situation thus demands external antioxidant supplementation obtained from either natural or synthetically prepared sources [9]. Several marketed drug molecules have also been ascribed as antioxidants [10] and are being extensively used in cardiovascular diseases.

The increased demand for antioxidants has oriented a great deal of present research towards the design and development of new chemical entities with potential antioxidant activity. This task of hoping for new molecules with improved activity potential has been extensively simplified by the quantitative structure-activity relationship (QSAR) methodologies [11]. The QSAR technique mathematically correlates the biological activity of the molecules with their various structural features that impart a distinct variation in the different physicochemical properties of the molecules. Thus, the QSAR technique provides an easy searching tool in the virtual screening procedure and has been widely adapted by different groups of researchers for identifying novel chemical entities. Garrido *et al.*[12] unified different families of antioxidants in a single model by employing the QSAR technique for a heterogeneous group of substances using TOPS-MODE descriptors to interpret their antioxidant activity in the form of bond contributions. Li *et al.*[13] performed QSAR analysis using 214 tripeptides using different sets of amino acid descriptors. The model signifies the different structural variants of the amino acid residues of antioxidative peptides. Several works related to the determination of antioxidant properties of various chemical entities (hydroxybenzalactones, benzothiophenes, chromone derivatives, oxazine derivatives, phenolic compounds, caffeic acid derivatives etc.) have also been performed by our group [14–17].

The coumarin derivatives constitute an essential natural source of antioxidants obtained from green plants belonging to the family Rutaceae and Umbelifferae. These molecules have been found to exert free radical scavenging activity in human tissue through a variety of mechanisms chiefly due to their structural equivalence to the flavonoids and benzophenones [18]. Further studies revealed that molecules with coumarin-like structure can inhibit formation of hydroxyl and hydrogen peroxide radicals during Fenton’s reaction by chelating with ferric ion [19]. They have also been reported to prevent ROS-mediated cell damage by inhibiting the xanthine-oxidase enzyme [20]. In the present work, a series of coumarin derivatives have been modeled for their antioxidant activity. The QSAR models developed in the present work employ different ligand-based strategies aiming to design new derivatives, bearing the coumarin nucleus, with potent antioxidant activity. The descriptor-based QSAR model developed here provides useful insight regarding the molecular pre-requisites for exhibiting potent antioxidant activity, while the pharmacophore [21] and the HQSAR [22] models constitute essential 3D query tools for identifying the useful pharmacophoric features and the fragment contributions of the molecules.

## Method and materials

### The dataset

**Fig. 1:**
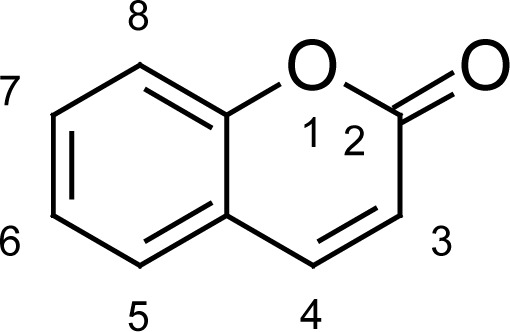
Parent coumarin nucleus analysed in the present study

In the present work, a series of coumarin derivatives were modeled for their antioxidant activity using efficient chemometric tools. The data were collected based on the results reported by three different groups of authors viz. Zhang *et al.*[23], Cavar *et al.*[24], and Panteleon *et al.*[25]. The dataset reported by Zhang *et al.*[23] comprised of a series of 13 novel 4-Schiff base-7-benzyloxy-coumarin derivatives while the work reported by Cavar *et al.*[24] comprised of 23 compounds belonging to the class of 4-methylcoumarins. The third dataset reported by Panteleon *et al.*[25] comprised of a series of nine spiropyrano-coumarin derivatives. The parent nucleus for all of the molecules is shown in Fig. 1. The data reported in all the papers were estimated using the same experimental protocol i.e., the ability to scavenge the 1,1-diphenyl-2-picryl-hydrazyl (DPPH) free radical. For the development of the pharmacophore models, the dependent variable IC_50_ (nM) (50% inhibitory concentration) of the molecules was used, while pIC_50_ [log_10_(1/IC_50_)] was used for the QSAR and HQSAR analyses.

### Development of QSAR models

Furthermore, descriptors belonging to different categories *viz.* spatial, topological, electronic, and structural were calculated for the development of QSAR models using Cerius 2 software [26]. For the development of the QSAR model, the whole dataset was divided into training and test sets comprising of 32 and 13 compounds, respectively. The splitting of the dataset was done based on the cluster analysis technique [27] so as to ensure that the training set molecules span the entire chemical space of the molecules under study. The training set molecules were thus employed for the development of the QSAR models while the test set was utilized for subsequent validation of the models. Three different QSAR models were thus built for the present work: (a) the descriptor-based QSAR model, (b) 3D pharmacophore model [21], and (c) fragment-based QSAR model developed using the hologram QSAR (HQSAR) technique [22]. The various QSAR models thus developed together with their validation strategies are detailed below.

#### Descriptor-based QSAR model

Two different chemometric tools (genetic function approximation or GFA and genetic partial least squares or G/PLS) [28, 29] were employed to determine the correlation between the activity of the molecules and the corresponding descriptors for the development of the descriptor-based QSAR models. The genetic function approximation technique was thus selected for variable selection while the linear regression technique and the partial least squares methodology were employed for model development. The GFA and the G/PLS models thus developed were validated based on different internal and external validation measures to ensure the predictive ability and the robustness of the developed models.

#### 3D pharmacophore model

The 3D pharmacophore model was developed using the discovery studio 2.1 software [30], based on the Hypogen module [21]. The pharmacophore refers to the spatial arrangement of molecular features that are responsible for the specific biological activity of the molecules, and the 3D pharmacophore model thus developed determines the essential structural attributes of the molecules for exhibiting the desired response. A 3D pharmaco-phore model was developed using conformers obtained from the *BEST* method of conformer generation based on the poling algorithm [31]. For the assessment of the quality of the generated pharmacophore hypotheses, cost functions [21] (represented in bits unit) were calculated during hypotheses generation. Additionally, the statistical significance of the pharmacophore models was determined using the Fischer validation test at the 90% confidence level and the values of the cost functions for the randomized and non-randomized models were compared. External validation was performed based on the activity prediction of the test set compounds that were mapped to the developed pharmacophore model, and the predictive ability of the model was analysed based on the value of predictive R^2^ (R^2^_pred_).

#### HQSAR model

Besides these, the HQSAR methodology [22] was also performed to determine the impact of the different molecular fragments towards their response parameter. A molecular hologram is a form of fingerprint encoding technique that unravels the importance of all possible molecular fragments which include linear, branched, cyclic, and overlapping features of the molecules. The HQSAR model was derived based on various combinations of fragment distinction and fragment generation parameters for each hologram length using the Sybyl 7.3 [32], and the selection of the best model was based on the maximum Q^2^ value at the optimum component number. The optimum number of components was also further checked based on the “5% rule,” which permitted an addition of a latent variable only when the addition resulted in an increment in the value of Q^2^ by 5% or more [33]. However, the chances of overfitting the developed model were reduced by limiting the maximum number of components to n/5 (n is the number of training set compounds). The final HQSAR model was thus obtained based on PLS analysis with an optimum component number based on the specific fragment distinction parameters, fragment size, and bin length. The predictive potential of the HQSAR model was analysed based on the activity prediction of test set molecules followed by calculation of the value of the R^2^_pred_ parameter.

### Validation of the developed models

The statistical significance of the models was assessed based on the value of LOO-Q^2^ (leave-one-out cross-validated squared correlation coefficient) which was calculated using the predicted activity data of each of the training set compounds that were deleted once in each of the cycles of LOO cross-validation. Furthermore, the proximity in the values of the predicted and observed activity data of the training set compounds was ascertained based on the calculation of the r_m_^2^ metrics [ $\bar{{\text{r}}_{\text{m}(\text{LOO})}^{2}}$ and $\Delta {\text{r}}_{\text{m}\left(\text{LOO}\right)}^{2}$] (Eqs. 1 and 2) [34, 35].

Eq. 1. $$\bar{r_{m}^{2}}=\frac{\left(r_{m}^{2}+r{'}_{m}^{2}\right)}{2}$$

Eq. 2. $$\Delta r_{m}^{2}=\left|r_{m}^{2}-r{'}_{m}^{2}\right|$$

Here, ${\text{r}}_{\text{m}}^{2}={\text{r}}^{2}\left(1-\sqrt{{\text{r}}^{2}-{\text{r}}_{0}^{2}}\right)$ and r'm2=r2(1-r2-r02). Squared correlation coefficient values between the observed and predicted activity values of the training set compounds with intercept (r^2^) and without intercept ( ${\text{r}}_{0}^{2}$) were calculated for the determination of ${\text{r}}_{\text{m}}^{2}$. For the calculation of the ${\text{r}}_{\text{m}}^{2}$ metric, the observed and the corresponding predicted activity data of the molecules was plotted along the X and Y axes, respectively. Subsequently, the value of r'02 was obtained by interchanging the axes and the r'm2 metric was calculated based on the value of r'02. Additionally, the external predictive potential of the developed models was judged based on the value of predictive R^2^ (R^2^_pred_) [36]. Besides the traditional metrics, the fitness between the observed and predicted activity values of the test set compounds was also assessed from $\bar{{\text{r}}_{\text{m(test)}}^{2}}$ and $\Delta {\text{r}}_{\text{m(test)}}^{2}$ parameters. Similarly, the overall predictive potential of the developed models in terms of both internal and external predictive ability was affirmed from the calculation of the overall predictive parameters, $\bar{{\text{r}}_{\text{m(overall)}}^{2}}$ and $\Delta {\text{r}}_{\text{m(overall)}}^{2}$. The descriptor-based QSAR models were also validated based on the randomization technique in order to ensure the robustness of the developed models. The randomization methodology employs permutation of the activity data keeping the descriptor matrix unchanged. In the present work, both process (involves scrambling of the activity data keeping the total descriptor matrix unchanged) and model (involves shuffling of the activity data with the model descriptors unaltered) randomization tests were performed at 90% and 99% confidence levels, respectively. The average squared correlation coefficient (R_r_^2^) calculated from the models developed using the permuted data matrices should be much lower than that of the original model R^2^, so as to reflect the existence of a true correlation for the developed models. The additional calculation of the ^c^R_p_^2^ parameter (threshold value = 0.5) checks for sufficient difference between the values of R^2^ and R_r_^2^[37].

## Results and Discussion

Three different QSAR models were built for the present work: (a) the descriptor-based QSAR model, (b) 3D pharmacophore model, and (c) hologram-based QSAR (HQSAR) model. The models thus developed could quantitatively determine the essential structural attributes imparting an optimum activity profile to the molecules under study. The descriptor-based QSAR model provides quantitative insight about the different molecular pre-requisites for exhibiting potential antioxidant activity. The pharmacophore model and the HQSAR model enable the detection of the different molecular features and the atomic fragments that are of utmost importance to impart optimum antioxidant activity to the coumarin derivatives. The structures of all the dataset molecules, together with their observed and predicted/calculated activity data, are listed in Table 1. Table 2 provides a summary of the results obtained from the three different methods employed in the present work for the development of QSAR models.

### Descriptor-based QSAR model

Among the four different models developed using the GFA and G/PLS techniques, the GFA spline model was selected as the most significant one based on the maximum values of the different validation parameters. The advantage of the spline function lies in its ability to determine a definite range for the descriptor value using a knot for the spline. A negative value with a spline term accounts for its zero contribution to the overall activity profile of the molecules, while a positive value of the spline term exerts its influence based on the sign assigned to its corresponding coefficient.

Eq. 3. $$\begin{array}{l} \text{pC}=-0.123+1.243\times \text{Atype}\_\text{O57}+0.010\times \text{JursPPSA}\_1-0.806\times \text{Atype}\_\text{C}25\\ +0.085\times^{2}\kappa +67.5\times <\text{Density}-1.18046>\\ {\text{n}}_{\text{training}}=32,\;\;\text{s}=0.352,\;\text{F}=66.98,\;{\text{R}}^{2}=0.928,\;{\text{R}}_{\text{a}}^{2}=0.914,\text{PRESS}=7.122,\\ {\text{Q}}^{2}=0.841,\;\bar{{\text{r}}_{\text{m }\left(\text{LOO}\right)}^{2}}=0.796,\Delta {\text{r}}_{\text{m}\left(\text{LOO}\right)}^{2}=0.050,\;{\text{R}}_{\text{pred}}^{2}=0.908,\bar{{\text{r}}_{\text{m}\left(\text{test}\right)}^{2}}=0.725,\\ \Delta {\text{r}}_{\text{m}\left(\text{test}\right)}^{2}=0.128,\;\bar{{\text{r}}_{\text{m}\left(\text{overall}\right)}^{2}}=0.813,\;\Delta {\text{r}}_{\text{m}(\text{overall})}^{2}=0.069 \end{array}$$

Here, *n_training_* and *n_test_* refer to the number of compounds in the training and test sets, respectively. Besides these, R^2^ and F refer to the determination coefficient and the variance ratio for the model at a specified degree of freedom (df), respectively. The threshold value for the majority of the validation parameters is 0.5 while the cut-off for the *Δr_m_^2^* parameter is 0.2. The values of all the statistical parameters being within the acceptable limit reflect the internal and external predictive potential of the developed model. The least possible deviations of the predicted activity data from the corresponding observed ones is further implied from the satisfactory values of all the *r_m_^2^* metrics. The value of ^c^R_p_^2^ (process randomization: 0.850, model randomization: 0.780), calculated based on the randomization tests, was much higher than the threshold value of 0.5 and thus ensured that the model was not just the mere outcome of chance.

Based on the standardized coefficients, the descriptors appearing in the above equation may be ranked as follows: (i) *JursPPSA_1*, (ii) *Atype_O57*, (iii) *Atype_C25*, (iv) *<Density – 1.18046>,* and (v) *^2^κ*. The *JursPPSA_1* descriptor refers to the partial positive surface area of the molecule which in turn indicates the sum of the solvent-accessible surface areas of all positively charged atoms (calculated using a sphere with a radius of 1.5Å to approximate the contact surface formed when a water molecule interacts with the considered molecules). A positive coefficient for this descriptor refers to an increment in the activity profile of the molecules with an increase in the value of this descriptor as seen in the case of compound nos. **2**, **6**, **9**, **10**, **11,** and **12**. Similarly, the low range values for the *JursPPSA_1* descriptor accounts for the reduced activity profile of compound nos. **16**, **19**, **20,** and **21**. The *Atype_O57* and *Atype_C25* are atom-centered descriptors [38] providing a measure of the hydrophobicity of the molecules due to the presence of fragments bearing an oxygen atom, in the form of phenol, enol, and carboxyl OH, and a tertiary carbon atom (R---CR---R), respectively. The positive coefficients of these descriptors signify their influence conducive to the antioxidant activity profile of the molecules. Compound nos. **9**, **10**, **11,** and **12** bearing higher order values for the two descriptors exhibit enhanced antioxidant activity. The spline function for the density descriptor refers to an increase in the activity of the molecules with an increase in the value of the *Density* descriptor above the knot of the spline i.e., 1.180, thus indicating a decrease in the volume of the molecules. Thus, compound no. **6** bearing a positive value for the spline term exerts a potent activity profile. The *^2^κ* index is a topological descriptor that refers to the degree of branching based on the count of two-bond fragments. The *^2^κ* index describes the molecular shape in relation to the linear graph. Additionally, the descriptor has been extended to take into account the size differences among the heteroatoms and carbon atoms in various valance states. A positive influence of this descriptor on the activity profile of the molecules signifies its favouring impact on their antioxidant potential. Thus, increased branching in case of compound nos. **6**, **9**, **10**, **11,** and **12** accounts for their improved antioxidant activity. In a nutshell, it may be inferred that increased branching with a subsequent decrease in the volume of the molecules, as well as an increase in the number of oxygen atom-bearing fragments favour their antioxidant activity profile. Additionally, molecules with increased solvent-accessible surface areas having a positive charge show potent antioxidant activity. Compound nos. **40**, **43,** and **44,** despite bearing higher order values for the *JursPPSA_1* descriptor, show moderate activity profiles due to the inappropriate values of the remaining descriptors. Again, the improved activity profile of compound no. **24** is chiefly attributed to the acceptable values for the *Density* descriptor and the atom type functions despite bearing inferior values for the *JursPPSA_1* descriptor.

### 3D pharmacophore model developed for the present work

For the development of the pharmacophore models, conformers were generated for 32 training set compounds using the BEST method of conformer generation. Ten hypotheses were generated based on the poling algorithm available within the *HypoGen* module of the Discovery Studio software (Table 3). The hypotheses were ranked based on the cost functions and the correlation coefficients. Fischer’s randomization test was performed at a 90% confidence level in order to ensure the fitness of the developed pharmacophore model. The results obtained for the randomization test for hypothesis 4 inferred that the average value of the total cost for the randomized models (201.030) was much closer to the value of the null cost (205.747) than that of the fixed cost (124.328). On the contrary, the total cost obtained for the non-randomized model (149.863) was much closer to the fixed cost, ensuring the robustness of the developed model. Besides this, a difference of 81.419 bits between the total cost and the null cost values for hypothesis 4 ensures the existence of 90% probability of true correlation. Additionally, the value of the configuration cost being much less than the stipulated value of 17, further adds to the acceptability of the developed model. Furthermore, the external validation of the model was performed by mapping the test set molecules of the developed pharmacophore, setting the maximum omit fit value at 1. The predictive quality of the models was assessed based on the value of R^2^_pred_ and the r_m_^2^_(test)_ metric and based on these parameters, hypothesis 4, with maximum values for all the external validation parameters (R^2^_pred_=0.705, $\bar{{\text{r}}_{\text{m}\left(\text{test}\right)}^{2}}=0.542$, Δr_m_^2^_(test)_ =0.002), was approved to be the best one.

**Fig. 2:**
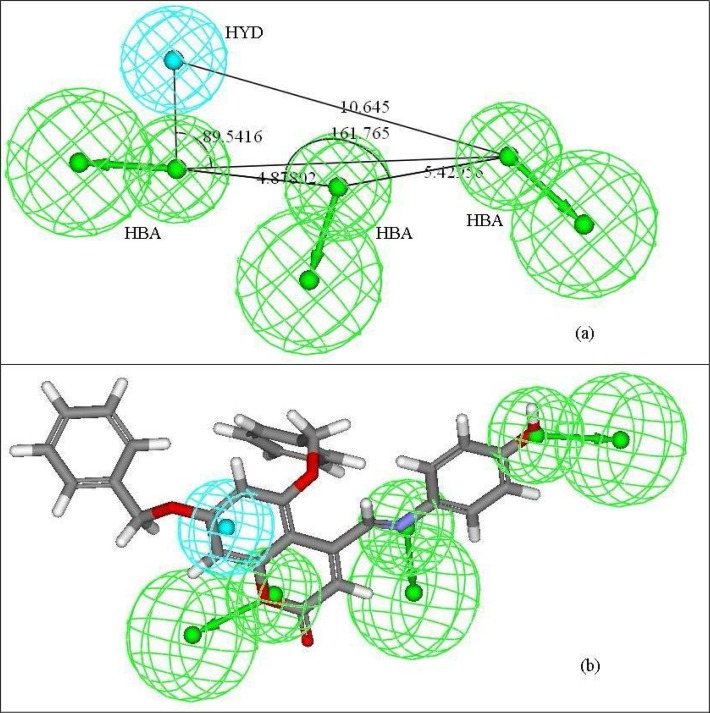
The pharmacophore obtained from hypothesis 4 (a) showing the distances among the different features and (b) mapping the most active compound (compound no. 10) to the developed pharmacophore. [Shown are the hydrophobic group (cyan) and hydrogen bond acceptor (green) features with vectors in the direction of putative hydrogen bonds]

The different chemical features displayed in hypothesis 4 include three hydrogen bond acceptor (HBA) features and a hydrophobic (HYD) feature. The three hydrogen bond acceptor features maintain an angle of 161.765Å among themselves where the central HBA feature lies at a distance of 4.878Å and 5.422Å from the remaining two HBA features. One of the HBA features makes an angle of 89.542Å with the HYD feature and the other HBA feature, while the HYD feature maintains a distance of 10.645Å from one of the HBA features (Fig. 2a) The direction of formation of the hydrogen bond between the antioxidant molecules and the free radicals was indicated from the vector direction for the HBA features. Again, regions favouring hydrophobic substituents are denoted by the hydrophobic features appearing in the 3D pharmacophore model. The importance of the hydrogen-bond acceptor groups for the optimum activity of the molecules indicates that they function by the mechanism of single electron transfer followed by deprotonation [39]. Mapping of the most active compound (compound no. **10**) with the developed pharmacophore indicated that the oxygen present in the pyran ring of the coumarin nucleus and the fused benzene ring constitute the hydrophobic and the hydrogen bond acceptor features, respectively, that are essential for the antioxidant activity profile of the coumarin derivatives (Fig. 2b). The hydrophobic nature of the benzene ring facilitates its interaction with nearby electron-rich free radicals. Additionally, the tertiary nitrogen linked to the coumarin nucleus through a methylene linkage constitutes a crucial fragment, capturing another hydrogen bond acceptor feature of the developed 3D pharmacophore model. The third hydrogen bond acceptor feature matches with the *para* hydroxy fragment of the substituent attached to the tertiary nitrogen atom substituted at C_4_ position of the parent nucleus. Similar observation has also been identified in the case of compound no. **9**. However, in the case of compound no. **6**, which lacks the *para* hydroxy fragment, the third hydrogen bond acceptor fragment matches with the oxygen atom of the nitro fragment attached at the *ortho* position of the substituent attached to the tertiary nitrogen atom substituted at C_4_ position. On the contrary, in the case of the spiropyranocoumarin derivatives (compound nos. **37**, **38**, **40**, **41**, **42**, **43,** and **44**), the hydrophobic feature matches with the fused benzene ring of the coumarin nucleus while the two hydrogen bond acceptor features capture the fragment of the coumarin nucleus and the hydroxyl substituent, respectively. Due to the inability of these compounds to match with the third hydrogen bond acceptor feature, the molecules exhibit a moderate activity profile. Thus, the reduced activity profile of compound nos. **16**, **17**, **27**, **29,** and **35** may be attributed to their inability to map with most of the essential pharmacophoric features, which in turn indicates the absence of the requisite atomic fragments in their molecular structures.

### HQSAR analysis

**Fig. 3:**
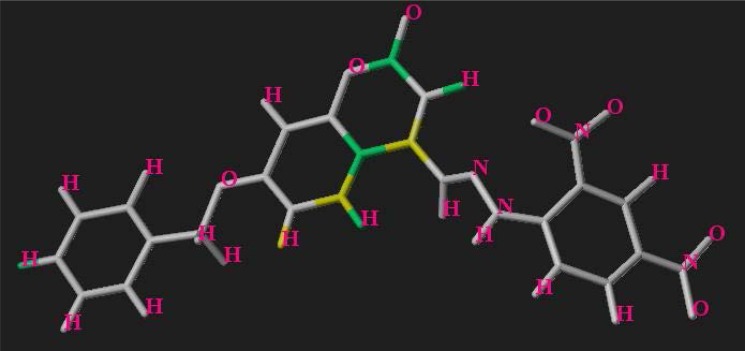
Contribution map obtained using the HQSAR technique based on compound no. 10 (see text for details)

The HQSAR analyses were performed with the training set molecules based on the optimization of different fragment features and the hologram length. The selection of the final PLS model was done based on the maximum value of Q^2^. The results of the HQSAR analysis are reported in Tables 4, 5, and 6. The analyses were first performed based on the training set molecules using the default fragment length with different combinations of the six fragment distinction features. Based on the values of maximum Q^2^ and minimum cross-validated standard error (SEcv), the best combination of the fragment features was selected [A (atom type) and D&A (donor and acceptor)]. The best fragment combination was then used to select the most suitable fragment size. The fragment size and the fragment combination thus optimized were utilized to select the significant hologram length. The fragment size, hologram length, and the optimum component number were selected based on the PLS analyses that yielded the lowest SEcv and the highest Q^2^. The component number was optimized using the 5% rule in order to reduce the noise and obtain a more robust model. The final model was obtained by repeating the analysis using the selected fragment contribution (A and D&A), fragment size (4–9), and hologram length (83) at an optimum component number of four. External validation of the model was performed based on the test set molecules with subsequent calculation of the external predictive parameter, R^2^_pred_. The significantly high value of this parameter (R^2^_pred_=0.704) indicated that the activity predicted for the test set molecules was well correlated to those of the observed ones. Furthermore, the *r_m_^2^* metrics were calculated to determine the proximity between the observed and predicted activity data. Acceptable values for all the statistical validation parameters reflect the predictive potential and the robustness of the developed model. Fig. 3 shows the contribution map for the developed HQSAR model referring to essential molecular fragments. The contribution map thus obtained indicates the degree of importance of the different fragments to the overall activity profile of the molecules based on their colouring pattern: (i) white color indicates an average contribution ranging from −0.097 to 0.102, (ii) red color denotes unfavorable contribution and ranges below −0.034, (iii) red-orange color also implicates similarly poor impact ranging between −0.034 to −0.020, (iv) yellow indicates a good contribution of 0.102 to 0.153, and (v) green signifies maximum contribution of 0.254 and above.

For the purpose of this discussion, contribution of the different fragments with respect to one of the most active compounds (compound no. **6**) has been shown here (Fig. 3). The green-coloured fragments, indicating their maximum contribution for governing the antioxidant activity of the molecules, include different fragments of the parent coumarin nucleus. The fragment C_2_ atom adjacent to the oxygen of the pyran ring bears the ketonic substituent and exerts maximum contribution. Besides this, the fused carbon between the benzene and the pyran rings has also been marked green, indicating the importance of the basic coumarin nucleus for the optimum activity of the molecules. The fragments contributing moderately to the activity profile of the molecules constitute C_2_ and C_5_ atoms of the parent coumarin moiety bearing the R_2_ and R_1_ substituents, respectively. Thus, compounds bearing all of the requisite molecular fragments exhibit potent antioxidant activity, while those lacking the essential fragments lie in the lower activity range.

Compound nos. **2**, **9**, **10**, **11,** and **12** bearing all of the indispensable molecular fragments and matching the vital pharmacophoric features, exhibit maximum antioxidant activity. However, compound nos. **16**, **17**, **27**, **29**, **34,** and **35** bear all of the essential fragments as indicated by the HQSAR analysis, although they lie in the lowest activity range due to their inability to map with all of the requisite pharmacophoric features.

## Overview and conclusion

**Fig. 4:**
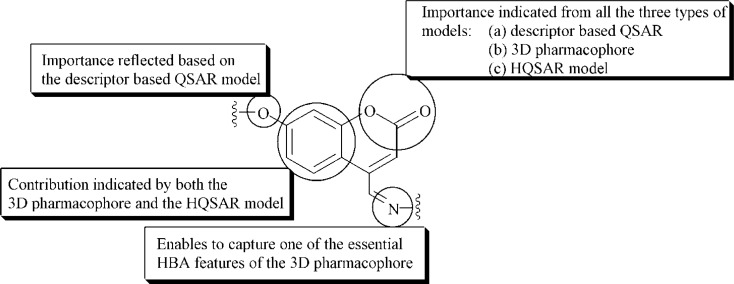
Schematic diagram showing different features at various positions favouring the antioxidant activity profile of the molecules obtained using different QSAR techniques

The present work deals with a series of coumarin derivatives that were modeled for their antioxidant activity based on their ability to inhibit DPPH free radicals. Attempts have been made to highlight the structural fragments that are of utmost importance for the molecules to execute their biological activity. The three different types of QSAR models developed here thus simplify the process of identifying the essential molecular fragments and thereby facilitate the selection of molecules exhibiting improved radical scavenging activity. The descriptor-based QSAR model was developed using two different chemometric tools which include the GFA and G/PLS methodologies based on both linear and spline options. Based on the different internal and external validation metrics, the G/PLS model developed using the spline option was selected as the best one. The model infers that compounds bearing a higher degree of branching, which subsequently leads to a decrease in their molecular volume, exhibit enhanced free radical scavenging activity. Furthermore, molecules with an oxygen atom-bearing fragment (−OH, phenolic fragment, =O etc.) as well as a tertiary carbon as substituents also lie in the higher activity range. Besides these, the pharmacophoric features responsible for the definite biological activity of the molecules was identified based on the 3D pharmacophore model developed using the *BEST* method of conformer generation. The best pharmacophore hypothesis, selected based on the correlation coefficient and the cost functions of the developed models, indicate that three hydrogen bond acceptor features and a hydrophobic feature lying at the specified distances from each other were chiefly responsible for the optimum antioxidant activity profile of the molecules. The importance of the hydrogen bond acceptor features thus corroborates well with the descriptor-based QSAR model, which reveals the significance of oxygen atom-bearing fragments to the overall activity profile of the molecules. Additionally, the contribution of the different molecular fragments to the overall activity of the coumarin derivatives was identified based on the HQSAR analysis. The contribution map for the HQSAR model revealed the importance of the parent coumarin nucleus for the optimal activity of the molecules. Fig. 4 provides an outline about the importance of the different molecular fragments as inferred from the three different models.

The results aptly match those of the pharmacophore analysis, which indicates the importance of the fused benzene ring and the oxygen atom of the pyran ring for capturing the hydrophobic feature and one of the HBA features, respectively. Thus, the different QSAR techniques applied in the present work correlate well with each other and provide a paradigm for searching for new molecules belonging to the class of coumarin derivatives that might exhibit potent antioxidant activity.

## Figures and Tables

**Tab. 1.** Structures of molecules under study along with their observed and calculated/predicted antioxidant activity data

**No.**	**Structures**	**Observed activity (*pIC_50_*with*IC_50_*in molar scale)**	**Calculated/Predicted activity^#^**
01*	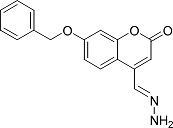	2.890	2.412
02	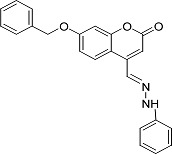	4.239	3.439
03*	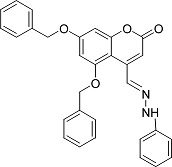	4.117	3.834
04	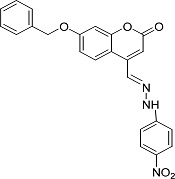	2.871	3.181
05*	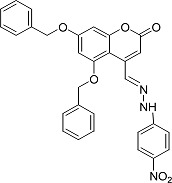	2.794	3.602
06	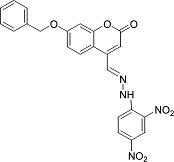	4.978	5.268
07*	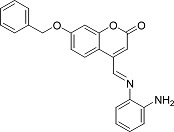	3.067	3.323
08	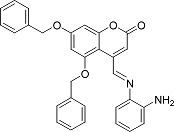	3.502	3.793
09	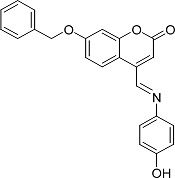	4.436	4.418
10	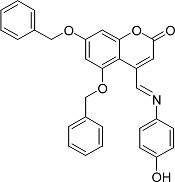	5.161	4.921
11	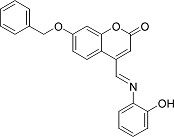	4.478	4.461
12	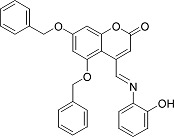	4.790	4.932
13	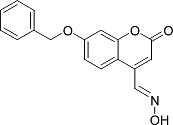	2.618	2.196
14*	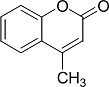	1.012	1.488
15*	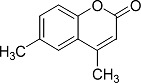	1.112	1.035
16	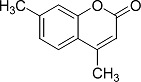	1.069	1.055
17	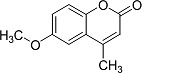	1.86	2.012
18*	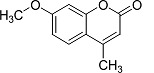	1.833	2.008
19	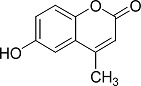	2.232	2.715
20	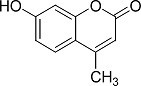	2.128	2.726
21	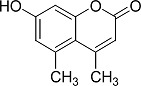	2.144	2.163
22	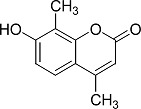	2.156	2.334
23	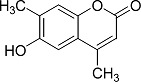	2.620	2.326
24	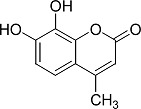	5.000	4.818
25	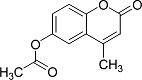	2.664	2.156
26	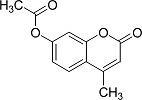	2.424	2.369
27	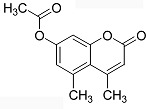	1.118	1.754
28*	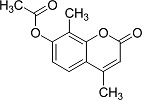	1.197	1.860
29	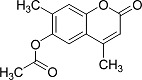	1.815	1.765
30	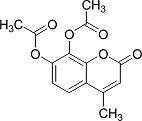	2.943	3.005
31*	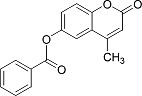	1.604	1.915
32*	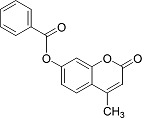	1.491	2.013
33*	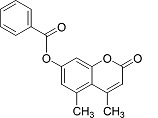	1.314	1.394
34	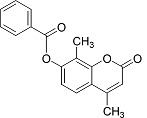	1.425	1.495
35	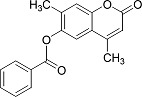	1.572	1.430
36*	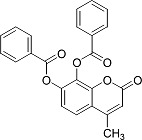	1.672	2.195
37	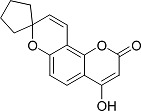	3.740	3.346
38	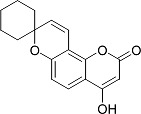	3.697	3.626
39*	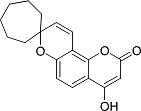	3.694	3.915
40	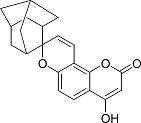	3.886	4.011
41	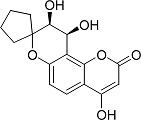	4.010	3.765
42	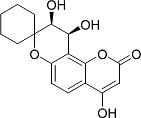	3.792	3.752
43	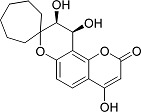	3.802	3.998
44	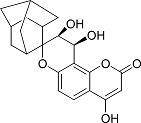	3.780	4.148
45	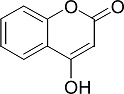	3.906	3.478

* Compounds constituting the test set;

# Activity calculated/predicted using the GFA spline model.

**Tab. 2.** Comparison of the statistical quality of all the developed models

**Statistical method**	**QSAR analysis**			

	**GFA**		**G/PLS**	

	**linear**	**spline**	**linear**	**spline**
Descriptors/Features/Fragments	Jurs-WNSA-2, Jurs-RNCS, Atype_O_57, Atype_C_1, Atype_H_51	Atype_O_57, Atype_C_25, Jurs-PPSA-1, ^2^κ, <Density-1.18046>	Atype_C_1, Atype_O_57, Atype_H_51, Jurs-WNSA-1	<2-Atype_O_57>, Atype_C_25, SC-0, <2.1913-AlogP98>

R^2^	0.906	**0.928**	0.882	0.875
Q^2^	0.848	**0.841**	0.830	0.807
$\bar{{\text{r}}_{\text{m}\left(\text{LOO}\right)}^{2}}$	0.785	**0.796**	0.759	0.731
Δr_m_^2^_(LOO)_	0.092	**0.050**	0.109	0.092
R^2^_pred_	0.833	**0.908**	0.859	0.784
$\bar{{\text{r}}_{\text{m}\left(\text{test}\right)}^{2}}$	0.621	**0.725**	0.673	0.581
Δr_m_^2^_(test)_	0.202	**0.128**	0.179	0.229
$\bar{{\text{r}}_{\text{m}\left(\text{overall}\right)}^{2}}$	0.742	**0.813**	0.745	0.701
Δr_m_^2^_(overall)_	0.080	**0.069**	0.142	0.171

**Process randomization (90%)**				

^c^R_p_^2^	0.780	**0.780**	0.638	0.603

**Model randomization (99%)**				

^c^R_p_^2^	0.830	**0.850**	0.880	0.873

**Statistical method**	**3D pharmacophore model**	**HQSAR analysis**		

Descriptors/Features/Fragments	HBA, HBA, HBA, HYDROPHOBIC	A/A & D		

R^2^	0.740	0.867		
Q^2^	–	0.525		
$\bar{{\text{r}}_{\text{m}\left(\text{LOO}\right)}^{2}}$	–	–		
Δr_m_^2^_(LOO)_	–	–		
R^2^_pred_	0.705	0.704		
$\bar{{\text{r}}_{\text{m}\left(\text{test}\right)}^{2}}$	0.542	0.489		
Δr_m_^2^_(test)_	0.002	0.270		
$\bar{{\text{r}}_{\text{m}\left(\text{overall}\right)}^{2}}$	–	–		
Δr_m_^2^_(overall)_	–	–		

**Tab. 3.** Results obtained from the Pharmacophore Hypotheses using the *BEST* method

**Hypothesis no.**	**Total cost**	**Correlation (R)**	**Features**	**Average cost function of 19 randomized models**	**R^2^_pred_**
1	147.433	0.877	HBA, HBA, HBD, HYD	191.388	0.560
2	147.727	0.875	HBA, HBA, HBD, HYD	196.083	0.523
3	148.363	0.870	HBA, HBA, HBA, HYD	199.159	0.579
**4**	**149.863**	**0.860**	**HBA, HBA, HBA, HYD**	**201.030**	**0.705**
5	150.028	0.864	HBA, HBA, HYD	202.851	0.535
6	150.442	0.863	HBA, HBA, HYD	204.110	0.545
7	150.725	0.855	HBA, HBA, HYD, RA	204.699	0.550
8	153.334	0.840	HBA, HBA, HBD, HYD	205.452	0.669
9	154.446	0.839	HBA, HBA, RA	205.920	0.589
10	157.896	0.824	HBA, HBA, HBD, HYD	206.667	0.602

Null cost: 205.747; Fixed cost: 124.328; Configuration cost (threshold: 17): 15.568

**Tab. 4.** HQSAR analysis for various fragment distinction using default fragment size (4–7)

**Fragment distinction**	**Q^2^**	**SE_cv_**	**R^2^**	**SE**	**LVs**	**Length**
A/C	0.437	0.916	0.519	0.846	1	151
**A/D&A**	**0.513**	**0.914**	**0.855**	**0.499**	**5**	**97**
A/B/C	0.430	0.921	0.519	0.847	1	401
A/B/H	0.461	0.944	0.788	0.592	4	199
A/B/C/H	0.411	0.952	0.529	0.852	2	97
A/B/C/D&A	0.454	0.902	0.538	0.830	1	401

**Tab. 5.** HQSAR analysis for the influence of various fragment sizes using the best fragment distinction (A/D&A)

**Fragment size**	**Q^2^**	**SE_cv_**	**R^2^**	**SE**	**LVs**	**Length**
4–8	0.489	0.937	0.864	0.484	5	199
**4–9**	**0.538**	**0.891**	**0.867**	**0.477**	**5**	**83**
5–8	0.490	0.936	0.861	0.489	5	199
5–9	0.529	0.900	0.866	0.479	5	83
6–8	0.484	0.941	0.865	0.482	5	307
6–9	0.537	0.892	0.867	0.477	5	83
7–8	0.496	0.931	0.874	0.464	5	307
7–9	0.507	0.903	0.797	0.580	4	83

**Tab. 6.** Selection of best model with less number of LVs using 5% rule

**LVs**	**Q^2^**	**SE_cv_**	**R^2^**	**SE**	**Length**	**% increase in Q^2^**
5	0.538	0.891	0.867	0.477	83	2.476
**4**	**0.525**	**0.886**	**0.808**	**0.564**	**83**	**32.242**
2	0.397	0.964	0.504	0.874	83	–

## List of abbreviations and notations

QSAR: Quantitative structure-activity relationship
HQSAR: Hologram quantitative structure-activity relationship
3D pharmacophore: 3 dimensional pharmacophore
IC_50_: 50% inhibitory concentration
DPPH: 1,1-diphenyl-2-picryl-hydrazyl
GFA: Genetic function approximation
G/PLS: Genetic partial least squares
LOO: Leave-one-out
R^2^: Squared correlation coefficient
s: Standard error of estimate
F(*df*): Variance ratio at specified degrees of freedom
Q^2^: Leave-one-out cross-validated squared correlation coefficient
R^2^_pred_: Predictive R^2^
